# In situ breast cancer surgeries in Sweden: lumpectomy or mastectomy?—a cost-effectiveness analysis over a 30-Year period using Markov model

**DOI:** 10.1186/s12962-023-00495-z

**Published:** 2023-11-10

**Authors:** Phu Duy Pham, Muchandifunga Trust Muchadeyi, Lars Lindholm

**Affiliations:** Department of Epidemiology and Global Health, Umeå University, 90185, Umeå, Sweden

**Keywords:** Breast cancer surgeries, Iumpectomy, Mastectomy, Cost-effectiveness analysis, Cost-effectiveness, Breast conserving Surgery, Follow-up irradiation

## Abstract

**Background:**

Breast cancer represents the most prevalent cancer among Swedish women. Although considerable research has investigated the cost-effectiveness of emerging innovative medical treatments for breast cancer, studies addressing existing surgical procedures remain scant. Therefore, this study aimed to evaluate the cost-effectiveness of three surgical procedures for in situ breast cancer treatment in Sweden: mastectomy, lumpectomy without irradiation, and lumpectomy with irradiation.

**Methods:**

A six-state Markov model with a 30-year time horizon was used to compare the cost-effectiveness of the three alternatives. Transition probabilities were based on a targeted literature review focusing on available evidence in Sweden and comparable contexts. Costs were estimated from both healthcare and societal perspectives, using patient data from the Swedish National Cancer Registry in 2020 (Cancerregistret). Health outcomes were quantified in terms of quality-adjusted life years (QALYs). Cost and health outcomes were then summarised into an incremental cost-effectiveness ratio (ICER) between competing strategies. A probabilistic sensitivity analysis (PSA) was conducted to address the uncertainties in the input parameters.

**Results:**

The results showed that compared to lumpectomy without irradiation, lumpectomy with irradiation yielded a “moderate” ICER per QALY gained of 402,994 Swedish Krona (SEK) from a healthcare perspective and a “high” ICER of 575,833 SEK from a societal perspective. Mastectomy proved to be the costliest and least effective of the three alternatives over a 30-year period. The PSA results further substantiated these findings.

**Conclusions:**

Our study demonstrated that lumpectomy with irradiation is “moderately” cost-effective compared with lumpectomy without irradiation. Nevertheless, extending this study by conducting a comprehensive budget impact analysis to account for the prevalence of in situ breast cancer in Sweden is prudent. These results imply that a costlier and less effective mastectomy should only be considered when lumpectomy options are infeasible. Further studies are needed to obtain more reliable parameters relevant to Sweden and to increase the consistency of the findings.

## Highlights

Lumpectomy and mastectomy have been used for a long time to treat breast cancer; however, their economic benefits have not been appropriately evaluated.

This study recommended lumpectomy as a “moderately” cost-effective surgical procedure for in situ breast cancer in Sweden.

For in situ breast cancer, mastectomy should be reserved for instances in which lumpectomy options are infeasible.

The findings of this study provide additional aspects for physicians and policymakers to consider in decision-making.

## Introduction

Breast cancer is the most common cancer type among women, accounting for the highest cancer incidence in 2020, with 7,534 new cases in Sweden [1]. Additionally, breast cancer is a leading cause of death worldwide, ranking third in the age-standardised mortality rate (11.4%), behind prostate and lung cancer [2].

In response to this persistent challenge, multiple innovative treatments have been developed for cancer, including surgery, radiotherapy, chemotherapy, and hormone therapy [3]. Surgical procedures, in particular, are integral to breast cancer management. In recent decades, lumpectomy, also known as breast-conserving surgery (BCS), has been developed and used along with the classic surgical procedure of mastectomy (or entire breast surgery) [4]. The choice between these procedures depends on several factors, including disease stage, patient condition, medical infrastructure, and clinical expertise [5, 6].

In Sweden, the proportion of women undergoing lumpectomy has increased significantly from 7% to 1980 to over 80% in 2021 [7]. Most of these lumpectomy cases are in early-stage breast cancer, including carcinoma in situ. This shift from mastectomy to lumpectomy correlates well with the nationwide breast cancer screening that has led to early breast cancer diagnosis among Swedish women, who are often young and value the cosmetic and psychological advantages of lumpectomy [6, 8]. Despite similar survival rates offered by both surgical methods, as validated through randomised controlled trials (RCTs) [9, 10], there remains a dearth of studies exploring their differential impacts on patient quality of life, especially over the long term [11, 12].

From a societal perspective, the total cost of breast cancer in Sweden in 2002 was estimated at 3 billion SEK, including nearly 900 million direct healthcare costs and over 2 billion indirect costs [13]. This shows the significant societal impact of breast cancer as a long-term, high-treatment-cost disease. A significant proportion of indirect costs is due to productivity losses resulting mainly from premature mortality (i.e., foregone labour market earnings that a person of working age who dies would have been expected to produce throughout their working life) [13]. This grave societal implications of breast cancer underscore the urgent need for the use of cost-effective interventions.

The number of health economic studies in Sweden has increased significantly. However, most health economic studies on breast cancer treatments focus mainly on cost-effectiveness analysis of new medical technologies, especially chemotherapy [14, 15]. Existing treatments, particularly surgical interventions, are frequently overlooked. A few studies [13, 16, 17] have separately investigated the cost and health outcomes of breast cancer and its treatments without extending them into full cost-consequence or cost-effectiveness analyses. With lumpectomy having superseded mastectomy over the past 30 years, a comparative cost-effectiveness analysis of these surgical options is notably lacking in Sweden.

Therefore, this study sought to fill this gap by performing a cost-effectiveness analysis comparing mastectomy and lumpectomy surgical procedures, focusing on in situ breast cancer in Sweden.

## Methods

### Study setting

This study analysed the cost-effectiveness of current surgical treatments for in situ breast cancer among Swedish breast cancer patients. Sweden is a high-income European country with a robust, predominantly tax-funded, social-based universal health care system [18]. In situ breast cancer, also known as carcinoma in situ of the breast, is coded as D05 in the International Classification of Diseases, Tenth Revision (ICD-10) classification system [19]. This classification system is widely used in national cancer registries, including the Swedish National Cancer Registry (Cancerregistret), which was used for cost estimation in this study [20].

The Swedish National Cancer Registry (Cancerregistret) dataset [20] incorporates information on the ICD-10 code, individual patient surgical and hospitalisation costs. Nevertheless, it lacks specific details on cancer stage and costs associated with surgical complications. To overcome this limitation and to clearly index patients into the model, an additional ICD-10 code (D05), representing carcinoma in situ of the breast, was used. Consequently, the study narrowed its focus to patients with in situ breast cancer.

In situ breast cancer corresponds to the earliest stage of breast cancer, where the malignant cells are still confined to their site of origin. The most prevalent form is ductal carcinoma in situ, characterised by cancerous cells residing in the lining of the breast milk duct, yet not extending beyond the duct into the surrounding breast tissue. Lobular carcinoma in situ, although less common, involves cancerous cells found in the breast’s milk-producing glands (lobules), again confined within the lobules.

### Model comparators

In situ breast cancer, often classified as stage 0, usually presents with appropriate resection margins and a ratio of tumour volume to size that enables effective management through various surgical interventions. These interventions include lumpectomy, optionally accompanied by follow-up irradiation or mastectomy. Lymph node removal is not usually needed with lumpectomy. Postsurgical initiation of hormone replacement therapy is an additional treatment consideration, regardless of the surgical approach employed. This study evaluated and compared three available surgical alternatives: mastectomy, lumpectomy without irradiation and lumpectomy with irradiation.

Mastectomy is a type of surgery that involves the removal of the entire breast. Mastectomy may be a treatment option at any stage of breast cancer, including in situ carcinoma depending on the doctor’s recommendation and the patient’s preferences [5, 6, 21]. This surgical method has existed for a long time and is still used in many settings [22].

Lumpectomy, also known as BCS, is a more recent surgical method developed in the early 1980s [23]. This method involves the removal of only a portion of the breast with cancerous cells or abnormal tissue. Follow-up irradiation is typically recommended for patients after a lumpectomy to limit breast cancer recurrence [23]. However, if no clinical value is expected, such as in the case of elderly patients or those with significant health problems, the surgeons may recommend against follow-up irradiation, given the potential for more harm than benefits [24]. The Swedish National Cancer Registry (Cancerregistret) dataset [20] utilised in this study did not stratify the types of follow-up irradiation. As a result, we considered irradiation as a general follow up method to lumpectomy for the purposes of this study.

### Perspectives and time horizon

In adherence to the guidelines provided by the National Board of Health and Welfare, this study was designed to capture the healthcare perspective and only included healthcare costs [25]. Additionally, a broader societal perspective was conducted to account for the indirect costs impacting society at large.

We estimated the difference between the average diagnosis age of in situ breast cancer patients derived from the Swedish National Cancer Registry data in 2020 (Cancerregistret) [20] (~ 58 years). Consequently, a 30-year horizon was chosen to reflect the lifetime approach used in this study.

### Model structure

A six-state Markov model, adapted from Pobiruchin et al. (2016) [27], was implemented in Microsoft Excel (model available upon request). The model [27], previously validated to the German context and built upon a previous model [28], offers several key benefits for our study. Firstly, it accurately reflected the prognosis following initial surgical treatment for our hypothetical cohort of Swedish in situ breast cancer patients, assuming successful treatment in their first surgery. Secondly, it was designed to utilise real-world data on breast cancer, aligning with our approach of calculating parameters using real-world evidence. Lastly, it differentiated between remission and cancer-free states, enabling us to assign unique utility values to these states.

**Fig. 1:**
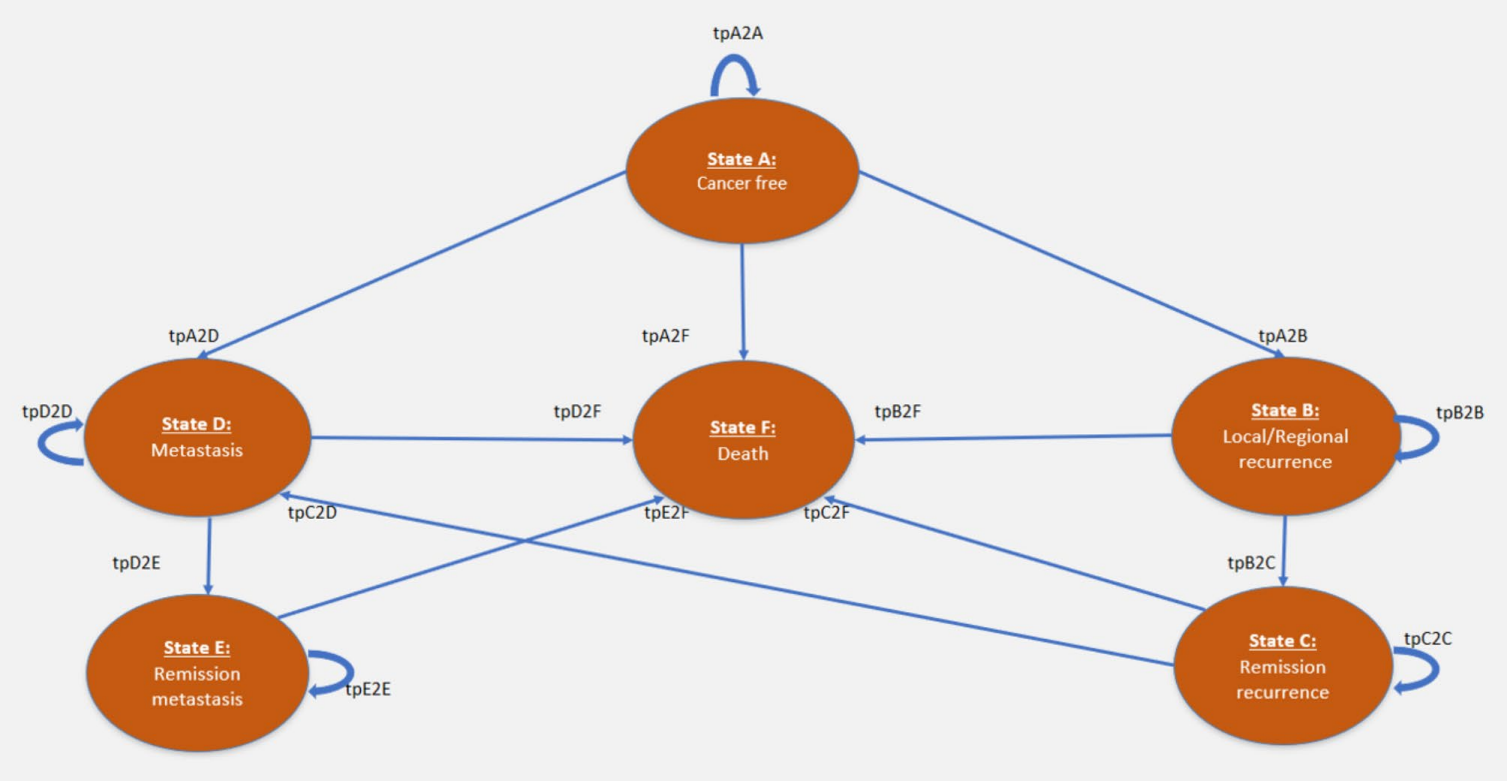
6-state Markov model of the cost-utility analysis comparing in situ breast cancer surgical procedures in Sweden

Figure 1 shows a schematic representation of the model structure. The model commences with all patients achieving a “cancer-free” status (State A) following their initial successful surgical treatment: mastectomy, lumpectomy with irradiation or lumpectomy without irradiation. Given the high success rates of early-stage breast cancer surgeries and similar outcomes of both mastectomy and lumpectomy procedures [9], the model did not consider complications from unsuccessful surgeries.

The remaining health states reflect the progression and prognosis of in situ breast cancer after successful initial treatment: “loco-regional recurrence” (State B), “metastasis” (State D), and “death” (State F). Ipsilateral breast cancer events were categorised as local or regional recurrence (State B), and contralateral breast cancer events as metastases (State D) [29]. Patients entering remission after experiencing recurrence or metastasis transitioned to the respective “remission states” (“remission after metastasis” [State C] and “remission after recurrence” [State E]). Importantly, these states are specific to patients who do not revert to their pre-remission disease state. This design eliminates the transitions from State B (or D) back to State A, thereby simplifying the model. Instances of repeated recurrence (second, third, etc.) were accounted for as a continuation in the respective disease state.

### Transition probabilities

Annual transition probabilities (tp) between states were calculated to represent movements between the six-state Markov model. Due to a specific focus on the Swedish context, a targeted review was conducted to gather evidence primarily from the Swedish or Scandinavian context. However, studies from other contexts were appropriately utilized when evidence from the Swedish context was not available. Notably, the three interventions differed primarily in their transition probabilities from cancer-free (State A) to loco-regional recurrence (State B) due to the difference in recurrence rates. Specifically, we assumed that, among the three event rates originating from the cancer-free (State A), only the recurrence rate from cancer-free (State A) to loco-regional recurrence (State B) can be significantly affected by the primary surgery type undertaken. In contrast, very long-term outcomes such as death from breast cancer and the onset of metastasis were considered not directly influenced by the surgery type following after achieving successful surgical intervention.

From this point on, we used the abbreviated names of the states and transition probabilities in the model to reduce repetition in the text. For example, the transition from cancer-free (State A) to loco-regional recurrence (State B) will be denoted tpA2B. Their full meanings can be found in Fig. 1; Table 1.

**Table 1:** Parameters of the model and their distributions

Parameter description	Name	Deterministic value	Evidences & Explanation	PSA Distribution*
Mean Age (at time entering the model)	age	58	Patient dataset from Swedish Cancer Registry data [20]	Normal (SD: 11.8)
General Swedish population Mortality rate	mor_gp	Varies on age	WHO (2021) [36]	NA
Annual transition probability of				
patient remaining at cancer free	tpA2A	Varies	= 1-tpA2B-tpA2D-tpA2F	NA
patient having loco-regional recurrence from cancer free	tpA2B	Varies	See Table 2	Beta (54.84, 944.16)
patient developing metastasis from cancer free	tpA2D	0.008	CI: 0.143 in 20-year followed up [17]	Beta (7.70, 991.30)
patient remaining at local/regional recurrence	tpB2B	0.770	= 1-tpB2C-tpB2F	NA
patient getting remission after suffering local/regional recurrence	tpB2C	0.165	CI: 0.763 in 8-year followed up [31]	Beta (164.52, 834.48)
patient dying from having local/regional recurrence	tpB2F	0.065	CI: 0.49 in 10-year followed up [33]	Beta (65.05, 933.95)
patient remaining remission after suffering local/regional recurrence	tpC2C	Varies	= 1-tpC2D-tpC2F	NA
patient developing metastasis after remission from local/regional recurrence	tpC2D	0.230	CI: 0.73 in 5-year followed up [34]	Beta (230.16, 768.84)
patient remaining at metastasis	tpD2D	0.447	= 1-tpD2E-tpD2F	
patient getting remission after suffering metastasis	tpD2E	0.375	CI: 0.609 in 2-year followed up [35]	Beta (374.33, 624.67)
patient dying from having metastasis	tpD2F	0.178	CI:0.86 in 10-year followed up [32]	Beta (178.31, 820.69)
patient remaining remission after suffering metastasis	tpE2E	Varies	= 1-tpE2F	NA
dying without breast cancer	tpA2F tpC2F tpE2F	Varies	= mor_gp	NA
remaining death	tpF2F	1.000		NA
Costs from healthcare perspective (in SEK)				
baseline cost—mastectomy	cam	87,670	Patient dataset from Swedish National Cancer Registry data [20] & Socialstyrelsen (2020) [41]	Gamma (656.50, 133.54)
baseline cost—lumpectomy without irradiation	cal_wo	27,655		Gamma (4,253.80, 6.50)
baseline cost—lumpectomy with irradiation	cal_w	50,352		Gamma (6,217.04, 8.10)
at State B, mastectomy	cbm	17,680		Gamma (26.70, 662.20)
at State B, lumpectomy (either with or without irradiation)	cbl	87,670		Gamma (42,749.59, 2.05)
at State D, all alternatives	cdm cdl_wo cdl	499,343	Lidgren et al. (2008) [40]	Gamma (1,386,844.91, 0.36)
Other costs from societal perspective				
informal care cost	infocare	10,003	Lidgren et al. (2008) [40]	NA
productivity gained (moving from mastectomy to lumpectomy in first 10-year followed-up)	prod	4,104	Norum et al. (1997) [12]	NA
Utility weights				
State A, mastectomy	uam	0.84	Norum et al. (1997) [12]	Beta (839.16, 159.84)
State A, lumpectomy (either with or without irradiation)	ual	0.87	Norum et al. (1997) [12]	Beta (869.13, 129.87)
State B	ub	0.78	Lidgren et al. (2007) [39]	Beta (778.22, 220.78)
State C	uc	0.81	Assumptions: Average of State A and B	Beta (808.69, 190.31)
State D	ud	0.69	Lidgren et al. (2007) [39]	Beta (684.32, 314.69)
State E	ue	0.76	Assumptions: Average of State A and D	Beta (761.74, 237.26)
Annual discounting rate				
costs (%)	cDR	3%	Edling and Stenberg (2003) [44]	NA
benefits (%)	oDR	3%	Edling and Stenberg (2003) [44]	NA

NA indicates not applicable; SD, standard deviation; SEK, Swedish krona

*PSA distributions presented with the format: “distribution type (alpha, beta)”

There was a significant difference in the loco-regional recurrence rate between lumpectomy with and without irradiation [17]. Consistent with other RCTs from the US and the UK [9, 10], the recurrence rate escalated over the years of follow-up, with a pronounced increase in the first 5 to 10 years, gradually diminishing thereafter [15]. Consequently, to account for this time-dependent transition probability, tpA2B, the follow-up time was divided into three periods: 0–5; 5–10 and 10 + years.

The incidence of loco-regional recurrence in lumpectomy with and without irradiation was collected from Wärnberg et al. (2014) [17], a Swedish RCT with a 20-year follow-up period. In the absence of a relevant Swedish study offering reliable data on mastectomy, the incidence of loco-regional recurrence in mastectomy was based on an RCT from the US [9]. To handle contextual differences, we calculated the incidence rate ratio between mastectomy and lumpectomy without irradiation (at 1.27) in the US study and applied this to our model.

The incidence rates retrieved from the literature were converted into annual transition probabilities using the formula *p = 1 – exp(-rt)* [30], where *p* is the transition probability, *r* is the rate, and *t* is the period of interest. Since the incidence rate had a unit of “per 1,000 person-years”, the value of *t* would be one year (to also correspond with the cycle length), and the value of *r* would be the incidence rate divided by 1000. The transition probabilities are listed in Table 2.

**Table 2:** Transition probabilities from State A (Cancer free) to State B (Loco-regional recurrence) (tpA2B)

**Incidence of local/regional recurrence by surgical methods and years of followed-up (per 1000 person-year) (Based on Fisher et al. (2002)** [9] **and Wärnberg et al. (2014)** [17])			
Years of followed-up	Mastectomy	Lumpectomy without irradiation	Lumpectomy with irradiation
0–5	56.46	71.70	36.90
5–10	26.77	34.00	24.30
10–30	7.09	9.00	8.30
Annual transition probabilities from State A to State B (tpA2B)*			
Years of followed-up	Mastectomy	Lumpectomy without irradiation	Lumpectomy with irradiation
0–5	0.055	0.069	0.036
5–10	0.026	0.033	0.024
10–30	0.007	0.009	0.008

*tpA2B were calculated from incidence rate of loco-regional recurrence using the formula: p = 1 – exp(-rt), recommended by Briggs et al. (2006) [30]

The model identified 16 unique transition probabilities (see Table 1). Seven transition probabilities (tpA2B, tpA2D, tpB2C, tpB2F, tpC2D, tpD2E, and tpD2F) out of the 16 were calculated based on cumulative incidence from clinical trials [9, 17, 31–35] and converted using the following formula: *p = 1 – exp(-rt)* [30]. Six transition probabilities were calculated by subtracting the other transition probabilities (tpA2A, tpB2B, tpC2C, tpD2D, tpE2E, and tpF2F) from 100% (see Table 1). The remaining three transition probabilities (tpA2F, tpC2F, and tpE2F) were considered similar to the probabilities of dying without breast cancer and were thus assumed to be equal to the age-specific mortality rate of the general Swedish population in 2020, derived from the World Health Organisation (WHO) website (2021) [36].

In addition, we accounted for the probability of dying from natural causes by adding the age-specific mortality rate of the general Swedish population [36] to all states except State F (death) in every year of follow-up.

### Cost estimation

Costs were estimated from both the healthcare and societal perspectives, in line with the recommendations from the Second Washington panel and the Swedish Dental and Pharmaceutical Benefits Agency (TLVAR) [37, 38]. All costs were valued in 2020 Swedish Krona (SEK), and were only accumulated in State A, State B, and State D, while remission states (State C and State E), and death state (State F) were assumed to incur no cost. The costs calculation were based on an individual-level patient cost dataset from the 2020 Swedish National Cancer Registry (Cancerregistret) [20] and other relevant supporting evidence [12, 13, 39, 40] (see Table 1). The cancer registry data are available upon request.

### Costs from the healthcare perspective

Costs from the healthcare perspective included the cost of surgery, hospitalisation, and follow-up irradiation (in case of lumpectomy with irradiation). Notably, adjuvant chemotherapy costs were included within the hospitalisation costs.

Baseline costs (before entering State A) accumulated during the first surgical treatment. Costs of surgery and hospitalisation were calculated based on the individual-level patient dataset from National Cancer Registry data in 2020 [20]. The dataset contained more than 5,000 patients undergoing lumpectomy and mastectomy. After restricting the analysis to only in situ cases, 96 (13.24%) have had a mastectomy, and 629 (86.76%) have had a lumpectomy.

Nevertheless, the dataset did not categorise patients according to whether they had lumpectomy with or without irradiation. To get around this, we identified and used the respective Swedish diagnostic-related group cost for lumpectomy without irradiation of 36,439 SEK in 2020 [41] to separate the two lumpectomy alternatives. Consequently, there were 240 (38.16%) lumpectomy without irradiation cases, and 389 (61.84%) cases with irradiation, among the total 629 cases of lumpectomy.

The average costs of mastectomy, lumpectomy without irradiation, and lumpectomy with irradiation were 87,670 SEK (SE: 3421.64), 27,655 SEK (SE: 424.02), and 50,352 SEK (SE: 638.59), respectively (see Table 1). While treatment costs varied across Sweden, these in-country cost variations were not significant. Additionally, a sensitivity analysis was conducted to account for these cost differences (see the section on sensitivity analysis).

At the loco-regional recurrence state (State B), the patients could receive different treatments based on their previous surgery. Recurrence after mastectomy was treated with adjuvant therapy, including radiation therapy and/or chemotherapy according to the recommended standard treatment guidelines. Radiation therapy costs, on average, 5,523 SEK [41]. The cost of hospitalization was assumed to be similar to a lumpectomy with irradiation, averaging 12,157 SEK per episode, based on the Swedish National Cancer Registry data [20]. Consequently, the total cost of a mastectomy alternative in this state was 17,680 (see Table 1).

Total mastectomy was usually recommended for patients with loco-regional recurrence after lumpectomy. Therefore, the total cost for each of the two lumpectomy alternatives at State B was assumed to be the same as that of a mastectomy episode, at 87,670 SEK.

Metastasis state (State D) accounted for the highest cost due to its complication. The cost of metastasis was assumed to be equal for all three surgical alternatives. We considered the most cost-effective treatment option based on a study by Lidgren et al. [40] at 425,174 SEK in 2005. This cost was calculated from a societal perspective, including the formal healthcare cost and informal care from family and friends. After adjusting for informal care costs and inflation, the healthcare cost for State D was 499,343 SEK (see Table 1).

The consumer price index (CPI) is the most widely used measure of inflation, which measures the overall change in consumer prices over time [42]. We applied the inflation calculator for Sweden provided by the WorldData [43] to convert all prices before 2020 to their relative amounts in 2020.

### Costs from the societal perspective

From the societal perspective, we considered costs associated with transportation, informal care, and productivity gains.

Transportation and informal care cost in the metastatic breast cancer state were estimated to be 8,350 SEK annually in 2005 in Sweden [40], and 10,003 SEK in 2020 after adjusting for inflation. We assumed that this cost was similar to the caregiver cost of the more extended hospitalisation options of a mastectomy and lumpectomy with follow-up radiation therapy. For lumpectomy without irradiation, the cost was assumed to be halved at 5,002 SEK (see Table 1).

Productivity gains were estimated based on a study from Norway [12]. When comparing mastectomy and lumpectomy in the first 10 years of follow-up, the productivity increased by 1% annually [12]. Based on this figure and the average salary of Swedish women in 2020 [36], annual productivity gained equalled 4,104 SEK per person (discounted) in the first 10 years for those receiving lumpectomy as their first surgery (see Table 1).

### Utility estimation

Health outcomes were estimated in terms of quality-adjusted life years (QALYs) as recommended by TLVAR [44]. To the best of our knowledge, no existing study in Sweden has reported QALY weights between lumpectomy and mastectomy. Therefore, we applied a study from the Norwegian context [12] to derive the QALY weights for health State A. Following successful treatment with mastectomy, lumpectomy without irradiation, or with irradiation, the respective QALY weights were 0.84, 0.87, and 0.87 [12] (see Table 1).

The QALY weights for “loco-regional recurrence state” (State B) and “metastasis state” (State D) were taken from a Swedish study [39] and established as 0.78 and 0.69, respectively. The QALY weight for “remission after loco-regional recurrence state” (State C) was assumed to be the average of States A and B. In contrast, the QALY weight for “remission after metastasis” (State E) was the average of States A and D (see Table 1).

### Discounting

The annual discount rate for both cost and QALYs was set at 3% as recommended by the general guidelines for economic evaluations from TLVAR [44].

### Cost-effectiveness analysis

The average cost-effectiveness ratios were computed for each surgical strategy by dividing the total 30-year cost by the corresponding QALYs for each procedure. To provide a more comprehensive comparison between the strategies, an incremental cost-effectiveness ratio (ICER) was calculated for each pair of competing strategies. The ICER was computed by dividing the costs difference between the two alternatives (e.g. A and B) by their corresponding difference in effects (QALYs in this study).

$$ICER= \frac{{Cost}_{A}- {Cost}_{B}}{{QALYs gained}_{A}- {QALYs gained}_{B}}$$

According to the recommended guidelines [45], all dominated strategies were excluded from the ICER computation. An intervention is considered dominated when it is less effective and more costly compared to an alternative intervention (“strongly dominated”) or the expected additional benefits (health outcomes) are derived at a higher marginal cost than necessary (“extendedly dominated”) [45].

Our study adhered to the guidelines set by the Swedish National Board of Health and Welfare [25], in which ICERs were segregated into four categories. Costs below 100,000.00 SEK per QALY gained were categorised as low, costs between 100,000.00 and 499,999.00 SEK as moderate, costs between 500,000.00 and 1 million SEK as high, and costs exceeding 1 million SEK (equivalent to US $108,652) per QALY gained as very high.

### Sensitivity analysis

Probabilistic sensitivity analysis (PSA) was performed to handle uncertainties. All inputs were varied according to the statistical distributions obtained from the patient-level dataset and relevant studies referenced (see Table 1). Utilising Excel VBA, the Monte Carlo simulation technique was used to randomly generate values from the joint distributions of costs, utilities, and transition probabilities across 1,000 iterations.

The probabilistic analysis results were presented using a cost-effectiveness plane (CE plane) and a cost-effectiveness acceptability curve (CEAC). The CE plane graphically depicts the outcomes of 1,000 random simulations, representing the uncertainty surrounding the deterministic cost-effectiveness value. The CEAC, on the other hand, represents the likelihood of one alternative being more cost-effective than another, in relation to a range of potential willingness-to-pay (WTP) thresholds.

## Results

Table 3 presents the results of the deterministic cost-effectiveness analysis (CEA) of the interventions under consideration. Following up on one in situ breast cancer patient in Sweden for 30 years after undergoing mastectomy, lumpectomy without irradiation, and lumpectomy with irradiation as the first surgical treatment resulted in an average of 11.21, 11.24 and 11.80 discounted QALYs, respectively.

**Table 3:** Costs, Utilities and ICER between alternatives in 30-year lifetime approach (discounted)

**A. Deterministic Results**						
**30-year followed up**	**Mastectomy** **(1)**	**Lumpectomy without irradiation** **(2)**	**Lumpectomy with irradiation** **(3)**	**1 vs. 2**	**2 vs. 3**	**1 vs. 3**
**QALY**	11.21	11.24	11.80	0.03	0.57	0.60
**Total cost (SEK)** **(Healthcare perspective)**	1,178,710	628,859	857,430	-549,852	228,572	-321,280
**Total cost (SEK)** **(Societal perspective)**	1,374,774	691,882	1,018,486	− 682,891	326,603	356,288
**ICER (Healthcare perspective)**				Dominated by 2*	402,994	Dominated by 3*
**ICER (Societal perspective)**				Dominated by 2*	575,833	Dominated by 3*
**B. Probabilistic Results**						
**30-year followed up**	**Mastectomy** **(1)**	**Lumpectomy without irradiation** **(2)**	**Lumpectomy with irradiation** **(3)**	**1 vs. 2**	**2 vs. 3**	**1 vs. 3**
**QALY**	10.56	10.67	11.01	0.11	0.35	0.45
**95%CI**	(5.52–13.75)	(5.61–13.80)	(5.71–14.50)			
**Total cost (SEK)** **(Healthcare perspective)**	1,342,074	826,459	1,008,674	-515,614	182,215	-333,399
**95%CI**	(630,402-1,745,438)	(343,150-1,257,338)	(459,686-1,391,397)			
**ICER (Healthcare perspective)**				Dominated by 2*	527,841	Dominated by 3*
**ICER (Societal perspective)**				Dominated by 2*	777,640	Dominated by 3*

CI indicates confidence interval; ICER, incremental cost-effectiveness ratio (unit: SEK/QALY gained); QALY, quality-adjusted life years; SEK, Swedish krona

*Mastectomy is dominated

From a healthcare perspective, the total costs per patient for the three alternatives averaged 1,178,710 SEK, 628,859 SEK and 857,430 SEK, respectively (see Table 3 A). As a result, the average costs per QALY for mastectomy, lumpectomy without irradiation, and lumpectomy with irradiation were 105,182 SEK, 55,970 SEK, and 72,646 SEK, respectively. Considering that mastectomy resulted in the lowest QALYs and the highest costs after the hypothetical 30-year follow-up, it was deemed to be dominated by two lumpectomy alternatives, and thus excluded from the ICER computations.

From a societal perspective, the related costs per patient for mastectomy, lumpectomy without irradiation, and lumpectomy with irradiation were estimated at 1,374,774 SEK, 691,882 SEK and 1,018,486 SEK, respectively (see Table 3 A). This resulted in a cost per QALY of 122,678 SEK, 61,579 SEK, and 86,292 SEK, respectively. Similar to the healthcare perspective analysis, mastectomy was dominated by the two lumpectomy alternatives.

When comparing lumpectomy without irradiation to lumpectomy with irradiation, the QALYs increased by 0.57 units for an individual patient over the 30-year follow-up period. Concurrently, the total costs increased by 228,572 SEK from a healthcare perspective and by 326,603 SEK from a societal perspective. As a result, the ICER for lumpectomy without irradiation compared to lumpectomy with irradiation was 402,994 SEK/QALY gained from the healthcare perspective. According to Swedish guidelines [25], this ICER falls into the “moderate cost per QALY gained” category. From a societal perspective, the ICER was slightly higher at 575,833 SEK/QALY gained, categorising it as “high cost per QALY gained” [25] (see Table 3 A).

### Sensitivity analysis results

Table 3B shows the results of the PSA. The sensitivity analysis further substantiated the deterministic finding that mastectomy was dominated by the two lumpectomy alternatives. The mean QALYs accrued after the 30-year follow-up after mastectomy were the lowest, and the mean costs of mastectomy were the highest among the three investigated alternatives.

**Fig. 2:**
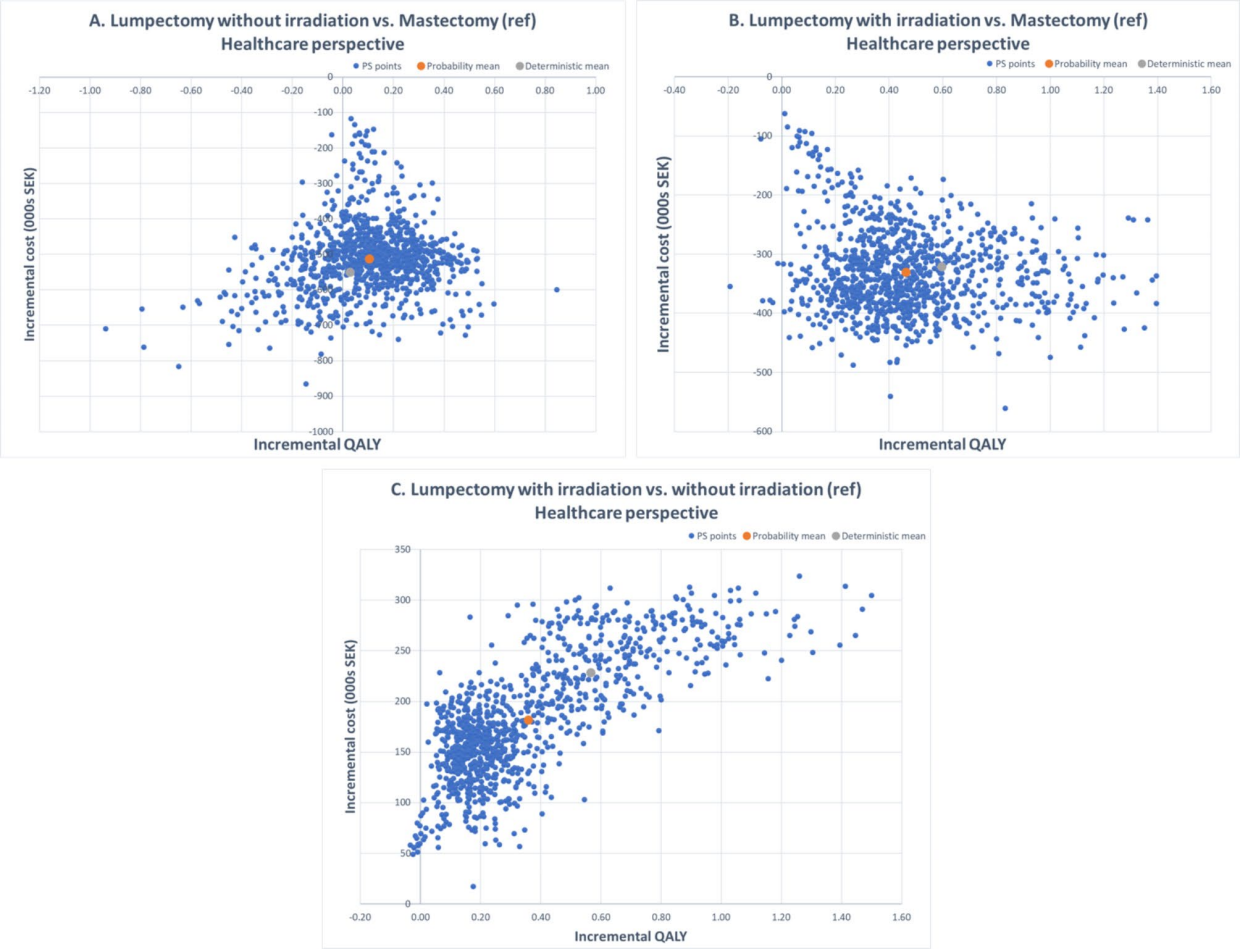
Cost-effectiveness planes comparing each pair of three alternatives under healthcare perspective

Figure 2 presents the CE plane for the PSA results. Notably, mastectomy consistently accumulated higher costs than the other two alternatives in every simulation, with all the simulated points for mastectomy located in the second and third quadrants of the CE plane.

When comparing mastectomy to lumpectomy without irradiation, most of the simulated points showed negative incremental QALYs (see Fig. 2A). In contrast, most simulated points indicated a positive incremental QALY when mastectomy was compared with lumpectomy with irradiation (see Fig. 2B).

When lumpectomy without irradiation was compared with lumpectomy with irradiation, the total costs consistently increased in all the simulated points. Likewise, the incremental QALY increased in most simulated points (see Fig. 2C).

**Fig. 3:**
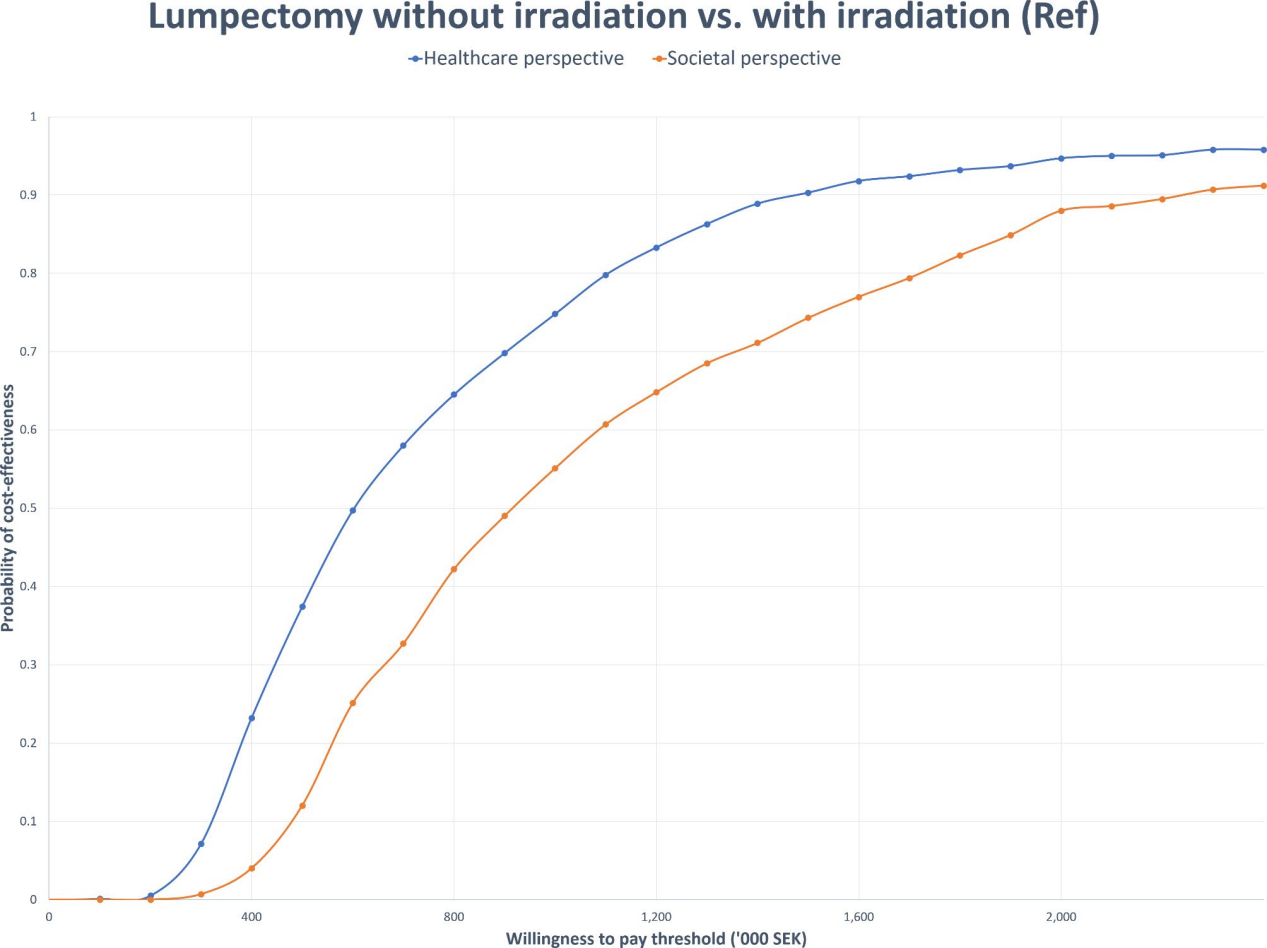
Cost-effectiveness acceptability curve (CEAC) comparing lumpectomy without and with irradiation

Figure 3 displays the CEAC for lumpectomy with radiation compared to lumpectomy without irradiation from both healthcare and societal perspectives. When compared with lumpectomy without irradiation, lumpectomy with irradiation only started exhibiting the slightest (> 0) probability of cost-effectiveness at a WTP threshold of 200,000 SEK per QALY gained from both healthcare and societal perspectives. From the healthcare perspective, increasing the WTP threshold from 300,000 SEK to 2 million SEK per QALY gained substantially increased the probability of cost-effectiveness of lumpectomy with irradiation from 22% to approximately 95% compared to lumpectomy without irradiation (see Fig. 3).

However, while the probability of cost-effectiveness of lumpectomy with irradiation compared to lumpectomy without irradiation increased with increasing WTP threshold from a societal perspective, it consistently fell short of the equivalent probability from a healthcare perspective. For example, at a WTP threshold of 2 million SEK per QALY gained, the likelihood of lumpectomy with irradiation being cost effective from a societal perspective was approximately 88%, compared to 95% from a healthcare perspective.

## Discussion

The present study aimed to evaluate the cost-effectiveness of current surgical treatments for in situ breast cancer in Sweden from both healthcare and societal perspectives using a lifetime horizon (30 years) approach. The results showed that mastectomy was more costly and less effective (dominated) than the two lumpectomy alternatives. Additionally, lumpectomy with irradiation was “moderately” cost-effective compared to lumpectomy without irradiation from the healthcare perspective, given the recommendation by the Swedish guidelines [25].

The study findings are consistent with those of Lidgren et al. [13], who suggested that the costs of mastectomy were higher than lumpectomy due to longer hospitalisation episodes. This cost difference between mastectomy and lumpectomy was even more prominent from the societal perspective, since the productivity gained was included in our study. Furthermore, the reimbursement based on the Swedish diagnosis related group (DRG) is higher for mastectomy than lumpectomy [41]. Smith et al. [46] also argued that there is a higher complication rate in invasive mastectomy surgery compared to lumpectomy, which might contribute to the higher costs of mastectomy in our applied dataset.

In contrast, Muñoz et al. [47] highlighted that lumpectomy with irradiation is the costliest treatment option due to higher physician fees plus radiation costs. However, this study only accounted for the baseline cost of hospitalisation, surgery, and irradiation, without considering additional costs related to complications, reconstructions, and informal care. Although the hospital dataset used in this study did not differentiate between a procedure with complications and without complications, we strongly assumed that this was adequately captured by the DRG prices and, therefore, can be considered a reliable estimate.

Lumpectomy with irradiation had the highest estimated QALY accruing over the 30-year period, with an increment in QALYs of 0.6 compared to the mastectomy option. Moreover, although we considered the same QALY weight for lumpectomy with and without irradiation at the beginning of the analysis, after a 30-year period, the difference was 0.57 in QALYs gained when comparing lumpectomy without irradiation to lumpectomy with irradiation. To the best of our knowledge, this study is among the first to studies compare the differences in QALYs between lumpectomy and mastectomy in Sweden using a lifetime approach. As a result, there were no other relevant studies to compare and corroborate our findings. However, a Turkish study [48] that applied a Markov model and a limited 10-year time horizon revealed QALYs gained of 0.58 when comparing mastectomy with lumpectomy, which can be considered similar to our study.

The major strength of the present study is that we applied a comprehensive six-state Markov model developed by Pobiruchin et al. (2016) [27]. The model closely resembled well the prognosis of early breast cancer patients after surgery. Additionally, the evidence used to derive the input parameters to populate the model was primarily based on Swedish registry data combined with relevant previous studies. Adding time dependency to the transition probability moving from State A to State B can mimic the real-world recurrence rate according to the length of the follow-up period, as suggested in previous RCTs [10, 17]. Finally, no assumptions were made regarding the 16 transition probabilities used in the model; to our knowledge, the best available evidence informed all the 16 transition probabilities (see Table 1).

Nevertheless, the study is also prone to some limitations. First, we applied a complex six-state Markov model, which requires more data sources to inform the input parameters. Consequently, not all input parameters were informed by sources from the Swedish context. While we acknowledge the impact of contextual and health system differences on costs and QALY weights, and ultimately the model results, this could only be partially avoided in cases in which we could not find relevant sources from Sweden.

Second, the QALY weights between lumpectomy and mastectomy in State A were based on a study from the Norwegian context [12]. The same study was conducted more than 20 years ago and can be considered outdated. Undoubtedly, with rapid technological advancement, improved training and skill of surgeons, better management of complications and healthcare becoming more patient centric, treatment outcomes are progressively improving. While this could have impacted the relevant transition probabilities, it was adequately addressed in the PSA.

Third, the incidence of loco-regional recurrence in mastectomy from an RCT from the US [9] was used to derive the transition probability (tpA2B) from the “cancer free” state (State A) to the “loco-regional recurrence” state (State B), due to the fact that there is no reliable evidence in Sweden for this. To address the limitation of borrowing evidence from another setting, we used the incidence ratio of recurrence between lumpectomy and mastectomy instead of using the actual incidence from a US study (see more in the Method section).

Finally, the individual patient dataset from the National Cancer Registry data in 2020 [20] used to derive the cost parameters has limitations. Since it is a population-based registry, it lacks details about the demographics and clinical characteristics of the patients. This limits our capacity to analyse other variables that may affect the choice of the surgical methods.

The net effect of lacking reliable evidence is the relatively wide ranges of QALYs, costs, and ICERs in PSA results (see Table 1). Despite these wide variations, the average value of 1,000 simulations from the PSA was similar to that of deterministic results.

## Conclusions

Our study demonstrates the dominance of lumpectomy over mastectomy. This reveals that more costly and less effective mastectomy should be considered only when lumpectomy options are infeasible. These scenarios could include instances where the in situ cancer is widespread (multicentric), the affected area is considerably large, or the BCS fails to remove the pre-cancerous cells entirely [49].

Moreover, our research indicates that lumpectomy with radiation is “moderately” cost-effective compared with lumpectomy without follow-up radiation therapy. Nevertheless, to comprehensively evaluate the economic impacts, we suggest a detailed budget impact analysis that factors in the prevalence of in situ breast cancer in Sweden.

The insights gained from this study have implications for future research and decision-making. First, this study is one of the first to compare the cost-effectiveness of surgical methods for in situ breast cancer in Sweden; further research should be facilitated to build more reliable parameters for the model. Based on the limitations and uncertainties of this study, future trials should be conducted to determine the necessary parameters for the model.

Second, future studies should be conducted in different contexts to derive more precise results, thus guiding the selection of the most appropriate treatment option within a specific context. We believe that the results of this study will contribute to the decision-making process considering lumpectomy with irradiation in in situ breast cancer treatment, especially where mastectomy is still the most common surgical method.

Finally, from our standpoint, health economic assessments remain crucial not only for new health technologies and treatments, but also for reassessing existing ones to evaluate whether they are worthy of continuation or if better alternatives are available. This consideration is especially pertinent given the variations in the context.

## Data Availability

The datasets used and/or analysed during the current study are available from the corresponding author on reasonable request.

## List of abbreviations

BCS: Breast conserving surgery
CEA: Cost-effectiveness analysis
CEAC: Cost-effectiveness acceptability curve
DRG: Diagnosis related group
ICER: Incremental cost-effectiveness ratio
PSA: Probability sensitivity analysis
QALY: Quality-adjusted life year
RCT: Randomized controlled trial
SEK: Swedish Krona
TLVAR: The Swedish Dental and Pharmaceutical Benefit Agency
WHO: World Health Organization
WTP: Willingness-to-pay
